# Improved Draft Genome Sequence of *Microbacterium* sp. Strain LKL04, a Bacterial Endophyte Associated with Switchgrass Plants

**DOI:** 10.1128/MRA.00927-19

**Published:** 2019-11-07

**Authors:** Mohammad Radhi Sahib, Piao Yang, Norbert Bokros, Nicole Shapiro, Tanja Woyke, Nikos C. Kyrpides, Ye Xia, Seth DeBolt

**Affiliations:** a Department of Horticulture, University of Kentucky, Lexington, Kentucky, USA; b Department of Horticulture, Al-Qasim Green University, Babylon, Iraq; c Department of Plant Pathology, The Ohio State University, Columbus, Ohio, USA; d Department of Energy Joint Genome Institute, Walnut Creek, California, USA; University of Maryland School of Medicine

## Abstract

We report here the genome assembly and analysis of Microbacterium strain sp. LKL04, a Gram-positive bacterial endophyte isolated from switchgrass plants (Panicum virgatum) grown on a reclaimed coal-mining site. The 2.9-Mbp genome of this bacterium was assembled into a single contig encoding 2,806 protein coding genes.

## ANNOUNCEMENT

Members of the genus Microbacterium have previously been isolated from a wide range of environments, including soils, marine ecosystems, air, and sewage, and from plants and insects (1–5). We report here information about the sequenced and assembled genome of the bacterial endophyte *Microbacterium* sp. strain LKL04, a Gram-positive actinobacterium, isolated from leaves of switchgrass plants grown on a reclaimed coal-mining site in western Kentucky (6).

Switchgrass samples were collected from the coal-mining site in July 2010. Leaf samples were cut into 1- to 1.5-cm-long segments, surface sterilized with a 20% bleach solution, and rinsed 5 times with autoclaved tap water. The surface-sterilized segments were incubated on tryptic soy agar (TSA) plates for 3 to 5 days at 26°C before the individual colonies were isolated and restreaked at least three times on new TSA plates (6). Single purified colonies were then isolated and grown at room temperature for 1 to 2 days in tryptic soy broth (TSB). A modified cetyltrimethylammonium bromide (CTAB) bacterial DNA isolation protocol (7; https://1ofdmq2n8tc36m6i46scovo2e-wpengine.netdna-ssl.com/wp-content/uploads/2014/02/JGI-Bacterial-DNA-isolation-CTAB-Protocol-2012.pdf) was followed to isolate the bacterial DNA for sequencing.

The genome of *Microbacterium* sp. strain LKL04 was sequenced at 212× coverage using Pacific Biosciences (PacBio) sequencing technology (8). A PacBio SMRTbell library was constructed and sequenced with the PacBio RS platform, generating 198,113 filtered subreads with an average read length of 3,930 bp ± 2,621 bp, totaling 778.5 Mbp. Reads were trimmed and assembled using Hierarchical Genome Assembly Process (HGAP) v.2.3.0 (9). The final genome assembly contains a single contig spanning the complete 2.922-Mbp length of the bacterial genome, with a GC content of 69.7%, which is characteristic of actinobacteria. The genome is precited to be circular.

**FIG 1:**
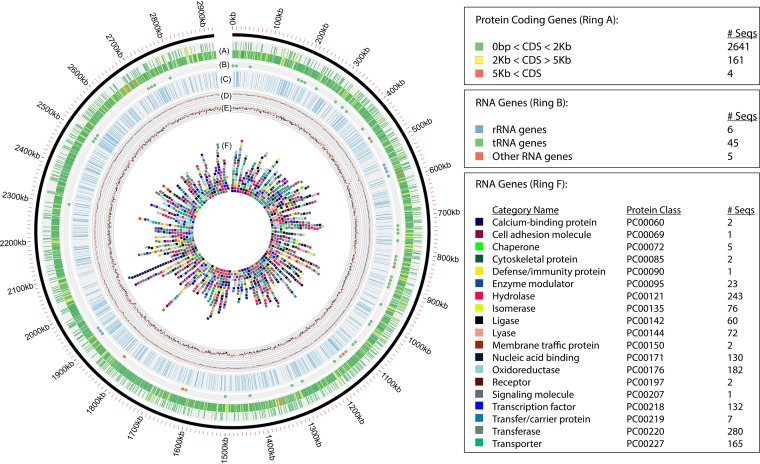
Circular representation of the LKL04 genome using Circos (25). The circles, from outside to inside, denote protein coding genes colored by size (A), RNA genes (B), transmembrane helix regions (C), GC content along a 1-kb window, with red lines indicating regions above the 69.7% genome average and black lines indicating regions below the genome average (D) GC skew, with red lines indicating a skew greater than zero and black lines indicating a skew less than zero (E), and genes annotated into distinct PANTHER protein classes (F). The repository for storage of scripts used to construct the figure can be found at https://github.com/nbo245/LKL04/tree/master/circos_plot.

Genes were identified using Prodigal v.2.5, followed by a round of manual curation using GenePRIMP, resulting in a total of 2,862 predicted genes (10, 11). From these, 2,806 predicted protein coding genes were translated and used to search the National Center for Biotechnology Information (NCBI) nonredundant, UniProt, TIGRFam, Pfam, Kyoto Encyclopedia of Genes and Genomes (KEGG), Clusters of Orthologous Genes (COG), PANTHER, and InterPro databases (12–18). For the remaining 56 genes, the tRNAScan-SE tool was used to further identify 45 tRNA genes, 6 rRNA genes, and 5 noncoding RNAs. For the noncoding RNAs, the RNA components of the protein secretion complex and RNase P were identified by searching the genome for the corresponding Rfam profiles using Infernal (19, 20). CheckM v.1.0.8, hosted on KBase, was used to estimate the completeness of the LKL04 genome (21, 22). Overall, the LKL04 genome returned a completeness score of 99.5% and a contamination level of only 0.67%. Using the PANTHER hidden Markov model (HMM) scoring tool pantherScore v.2.1, the protein sequences were further mapped against the PANTHER HMM database v.14.1 to functionally annotate the LKL04 genes and query for significantly overrepresented genes (23). Default parameters were used for each software program, unless otherwise specified. Selected annotations and genome characteristics are shown in Fig. 1. Additional gene prediction analysis and manual functional annotation were performed within the Integrated Microbial Genomes (IMG) platform developed by the Joint Genome Institute (Walnut Creek, CA) (24).

### Data availability.

The whole-genome sequence has been deposited in DDBJ/EMBL/GenBank under the accession no. PRJNA322991. Original forward and reverse sequencing reads can be retrieved from NCBI under SRA accession no. SRR4232145 and SRR4232146. The associated sequence data can also be found at the Joint Genome Institute (JGI) portal with the IMG taxon identifier (ID) 2667527218 (https://genome.jgi.doe.gov/portal/MicspLKL04/MicspLKL04.info.html) or at https://www.ncbi.nlm.nih.gov/Taxonomy/Browser/wwwtax.cgi?id=912630. Scripts used to construct Fig. 1 can be found at https://github.com/nbo245/LKL04/tree/master/circos_plot.
